# Monitoring Wound Healing With Contactless Measurements and Augmented Reality

**DOI:** 10.1109/JTEHM.2020.2983156

**Published:** 2020-03-30

**Authors:** Virginia Mamone, Miriam Di Fonzo, Nicola Esposito, Mauro Ferrari, Vincenzo Ferrari

**Affiliations:** 1 Department of Information EngineeringUniversity of Pisa 9310 56122PisaItaly; 2 EndoCASCenter for Computer-Assisted Surgery56124PisaItaly; 3 Department of Translational Research and New Technologies in Medicine and SurgeryUniversity of Pisa 9310 56122PisaItaly

**Keywords:** Augmented reality, wound healing assessment, non-invasive measurements, chronic wound, non-invasive, non-contact, miniaturized projectors, doctor-patient communication

## Abstract

Objective: This work presents a device for non-invasive wound parameters assessment, designed to overcome the drawbacks of traditional methods, which are mostly rough, inaccurate, and painful for the patient. The device estimates the morphological parameters of the wound and provides augmented reality (AR) visual feedback on the wound healing status by projecting the wound border acquired during the last examination, thus improving doctor-patient communication. Methods: An accurate 3D model of the wound is created by stereophotogrammetry and refined through self-organizing maps. The 3D model is used to estimate physical parameters for wound healing assessment and integrates AR functionalities based on a miniaturized projector. The physical parameter estimation functionalities are evaluated in terms of precision, accuracy, inter-operator variability, and repeatability, whereas AR wound border projection is evaluated in terms of accuracy on the same phantom. Results: The accuracy and precision of the device are respectively 2% and 1.2% for linear parameters, and 1.7% and 1.3% for area and volume. The AR projection shows an error distance <1 mm. No statistical difference was found between the measurements of different operators. Conclusion: The device has proven to be an objective and non-operator-dependent tool for assessing the morphological parameters of the wound. Comparison with non-contact devices shows improved accuracy, offering reliable and rigorous measurements. Clinical Impact: Chronic wounds represent a significant health problem with high recurrence rates due to the ageing of the population and diseases such as diabetes and obesity. The device presented in this work provides an easy-to-use non-invasive tool to obtain useful information for treatment.

## Graphical abstract

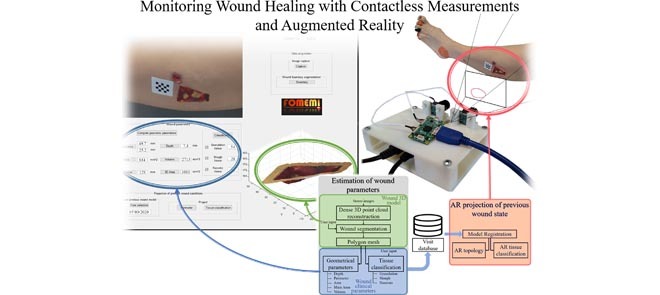

## I. Introduction

Chronic wounds, including vascular ulcers, pressure sores, and diabetic foot ulcers, result from a wound that, due to different pathological factors, failed to complete the physiological healing process and does not recover within three months [1]. These conditions represent a significant health problem; only in the U.S., about 6.5 million patients suffer from chronic wounds [2], with a great prevalence in adults over 65 years of age. Due to the ageing of the population and the spread of diseases such as diabetes, obesity, and vascular problems, the incidence of chronic wounds is expected to increase significantly [3].

Adequate monitoring of wound condition is crucial for guiding treatment decisions, especially for chronic wounds that may not follow the classical sequence of healing events [4]. In this regard, wound healing assessment methods can be distinguished into manual and computer-assisted methods. The former are the most widely used in nowadays clinical practice, which still relies on visual inspection and manual techniques to assess changes in size and tissue properties over time [5]. In the recent literature, computer-aided methods have been researched to provide objective evaluation parameters, minimizing the invasiveness for the patient. However, only two of these devices are currently being marketed. Following is an examination of manual techniques for wound healing assessment, an insight into the features of the commercially available devices, and a survey of the recent related literature. Finally, we will highlight the innovative functionalities of the device and discuss its usefulness in improving doctor-patient communication.

The most commonly measured parameters are: wound volume, area, perimeter, maximum depth, and tissue composition in the wound area (granulation, slough, and necrosis) [6]. The simplest manual technique to evaluate the wound surface area is derived from linear dimensional measurements and uses a ruler, but it only provides an approximation based on a regular shape model, for example a rectangle (length $\times $ width), or an ellipse (length $\times $ width $\times\,\,0.785$). Acetate tracing is a more accurate method, especially for complex shapes: it employs a transparent square sheet across the wound surface to trace its outline with a pen. The wound area is then determined by measuring the number of squares within the circumscribed area [7]. The maximal wound depth is estimated by inserting a cotton tip in the deepest recess of the ulcer. This position is difficult to find and the practice can cause severe pain to the patient. As to the wound volume, there are no accepted standard measurement techniques at present. Usually, volume is calculated by multiplying the maximal wound depth by the area (with large estimation errors). The volume may also be inferred by filling the wound with saline [8], but this method is not feasible for wounds located in inconvenient positions, and the measurement contains errors due to the presence of wound exudate [6]. Tissue composition analysis is commonly performed visually by a clinician by associating each area of the wound with a label in a red-yellow-black scale. The colors on this scale correspond to the dominant colors of the main tissues in the wound: red for granulation, yellow for slough, and black for necrotic tissue [9]. However, this assessment is still subjective and time-consuming.

In addition to their invasive nature, the manual methods described offer poor accuracy and reliability due to subjective interpretation and significant inter-observer variability.

To overcome the drawbacks of traditional methods, new devices have been proposed which provide a 3D reconstruction of the ulcer through non-contact techniques [10], [11]. Such measurement devices can be based on stereophotogrammetry techniques or laser scanner. The first category includes the solution proposed in [12], where the authors used multiple view geometry algorithms to generate a 3D model of the wound on which physical measurements are taken, while the laser scanner technology, to the best of the author’s knowledge, is employed in all the commercially available devices for wound assessment. Silhouette Star by Aranz Medical provides morphological measurements without any reconstructed 3D wound model. eKare inSightⓇ is a portable 3D measurement device for wound assessment, which combines an iPad and a Structure Sensor by Occipital to determine depth by using an infrared projector and camera system [13]. The details of Silhouette Star and eKare are reported in Table 1 [14], [15]. Accuracy in the estimation of a parameter denotes the deviation of the measured value from the true value and is expressed as:

\begin{equation*} Accuracy~\% =\frac {\left [{ measured~value-true~\mathrm { }value }\right]}{true~value}\cdot 100.\tag{1}\end{equation*}

Precision is expressed as coefficient of variation (CV). It measures the statistical variability of the measurements and is calculated as the ratio of standard deviation $(\sigma)$ to the mean value $(\bar {x})$ module:

\begin{equation*} CV=\sigma \mathrm {/}\left |{ \bar {x} }\right |.\tag{2}\end{equation*}

Data on inter-rater reliability are given as intra-class correlation coefficient (ICC).

**TABLE 1:** Ekare and Silhouette Specifications

		eKare	Silhouette
Accuracy	Area	2.55%	0.3%
	Perimeter	2.63%	0.01%
	Depth	2.87%	4%
	Volume	3.46%	−2.5%
Precision	Area	2.9%	1%
	Perimeter	11%	1%
	Depth	19%	2%
	Volume	3.3%	2%
Capture to measurement time		38.87’s	120’s
Inter-rater	Area	0.999	
reliability	Length	0.997	
	Width	0.995	
	Depth	0.649	
	Volume	0.696	

Data on Silhouette Inter-rater reliability are not available.

Recently, new low-cost technologies have been developed, such as a smartphone app [16] and a Microsoft Kinect sensor [17]. The former is a mobile platform that gives a sparse 3D model of the wound by using SLAM algorithms. This solution requires moving the smartphone around the ulcer to obtain the 3D reconstruction and the measurements. Although the method is easily accessible even in non-clinical settings, it is susceptible to errors and inter-operator variability, requiring the user to play an active role in the acquisition of the 3D model. The device proposed in [17] is based on Kinect v2 Time of Flight principle to estimate wound perimeter, area, and volume.

In our work, we developed a prototype that, in addition to estimating morphological parameters, offers AR functionalities, mainly to improve doctor-patient communication. Clinician communication skills are crucial for building a trustworthy doctor-patient relationship that contributes to therapeutic success. Patients who have a better understanding of their ailment increase their compliance to the doctor advices [18], [19]. Good communication has a whole series of positive effects on the patient’s psychology, mental health, tolerance power [20], but not all doctors are naturally gifted with it. Furthermore, wound progression can be evaluated through objective data, and a direct transfer of such information to the patient can overcome any subjective interpretation of the speaker (the doctor) and of the listener (the patient). For this reason, we integrated AR techniques to facilitate doctor-patient communication, as already demonstrated for other diseases. In their work, Wu *et al.* [21] used AR technology for the preoperative management of complex neck fractures, making doctor-patient communication simpler and more accurate. Touati *et al.* provide patients with a tablet to be used as an AR mirror to get a preview of their cosmetic dentistry surgery [22]. Augmented reality has also been widely used in rehabilitation to make the exercises more intuitive and alleviate monotony [23]. In this work, augmented reality is achieved through a miniaturized projector. Compared to the widely used head-mounted display, this projector allows its users to overcome ergonomic issues mainly related to the wearability of the devices and their perceptual limits [24]. A drawback of projector-based AR is the parallax effect that is caused every time the AR information is related to a non-exposed surface [25], which is not an issue in the case of wounds assessment.

Some recent works have positively evaluated the projector as a powerful tool to provide AR information: Gavaghan *et al.* presented a portable projector-based AR device for the visualization of navigation data in a surgical scenario [26] and Mewes *et al.* developed an AR system to visualize pre-planned paths and risk structures directly on the patient inside the MRI bore [27].

The aim of our work is to identify guidelines for the development of versatile and complete devices capable of obtaining both wound geometric parameters and tissue classification, and AR functionalities that provide an immediate feedback on the ulcer status in support of doctor-patient communication.

## II. Materials and Methods

### A. Device Overview

The AR wound monitoring device consists of a pair of Leopard Imaging LI-OV580 stereo cameras, size 26 mm $\times\,\,18$ mm $\times\,\,28$ mm each, and a Philips PPX4010 pico-projector, size 68 mm $\times\,\,66$ mm $\times\,\,22$ mm. The camera resolution is 2208 $\times\,\,1242$ @ 15 fps, which gives horizontal and vertical fields of view of 80° and 54°, respectively. The main information from the camera data sheet is reported in Table 2.

**TABLE 2:** LI-OV580 Stereo

Resolution ($\text{C}_{\mathrm {H}} \times \,\,\text{C}_{\mathrm {V}}$)	$2208\times 1242$
Frame rate	15 fps
Vertical field of view (FOV_V_)	80°
Horizontal field of view (FOV_H_)	54°

Technical data of the LI-OV580 stereo cameras. The horizontal and vertical fields of view are obtained from the nominal ones considering the sensor crop at the selected resolution.

**FIGURE 1.**
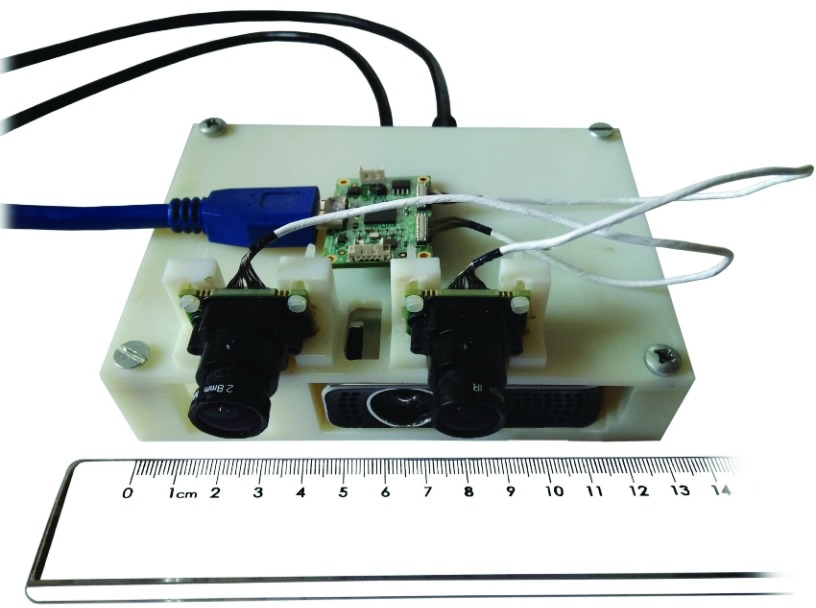
Wound healing assessment device prototype.

As shown in Fig. 1, the hardware is assembled on a printed support ensuring the stability of the reciprocal pose of the components. The cameras are mounted in parallel configuration with a baseline b = 45 mm. The working distance is set between 15 cm and 35 cm taking into account clinical needs. The system requires the application, on the patient limb, of a registration pattern consisting in an adhesive patch or on a semi-permanent tattoo depending on the clinical case. The pattern provides a reference for the alignment of models acquired over time and is essential for AR functionalities.

#### 1) Camera Stereo Calibration

For the proper operation of the device, both the stereo camera and the projector are calibrated in terms of intrinsic and extrinsic parameters, to obtain an accurate 3D reconstruction of the limb surface.

Camera stereo calibration involves intrinsic calibration, which gives the image formation parameters for each camera, and then extrinsic calibration, returning the roto-translation matrix for changing the reference system to the reference camera (the left one). Stereo calibration is performed in C++ language using OpenCV libraries with a $6\times5$ checkerboard pattern, 10 mm square size.

#### 2) Camera-Projector Calibration

Camera-projector calibration is required to precisely re-project the AR 3D information, acquired with the stereo camera, over the patient limb.

The projector has been calibrated against the reference camera by using the method proposed by Falcao in [28]. The projector pose and intrinsic parameters are derived by projecting a 15 $\times\,\,8$ digital checkerboard pattern with 80 pixel square size on a plane with different positions-orientation. The plane is defined by a second 4 $\times\,\,3$ checkerboard pattern with 10 mm square size and is used to get the 3D position of each projected corner by applying ray-plane intersection.

### B. Software Overview

**FIGURE 2.**
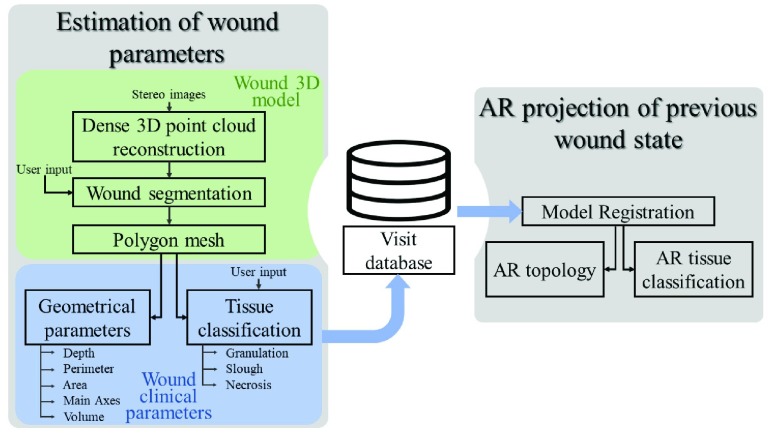
Software layout and system functionalities.

The block diagram in Fig. 2 illustrates the layout of the software and the relationships among its modules. The two main blocks, “Estimation of wound parameters” and “AR projection of previous wound state”, handle the main functionalities of the system, i.e. wound healing assessment through the monitoring of clinical parameters and through AR visualization of wound evolution, respectively. The first sub-block, the “Wound 3D model”, processes the stereo image pair through a stereo-photogrammetric 3D reconstruction algorithm that produces a dense point cloud of the wound surface and surrounding skin. The point cloud is then converted into a polygon mesh that constitutes the input to the next functional block, “Wound clinical parameters”. This block handles the estimation of morphological parameters and the classification of wound tissues. The data of each patient examination is stored and identified by patient ID and date. The “visit database” is the link to the last block that allows for a visual evaluation of the wound progress over time; the AR functionality is obtained by re-projecting the previous wound contours and/or tissue classification directly onto the patient’s skin.

#### 1) Wound 3D Model

**FIGURE 3.**
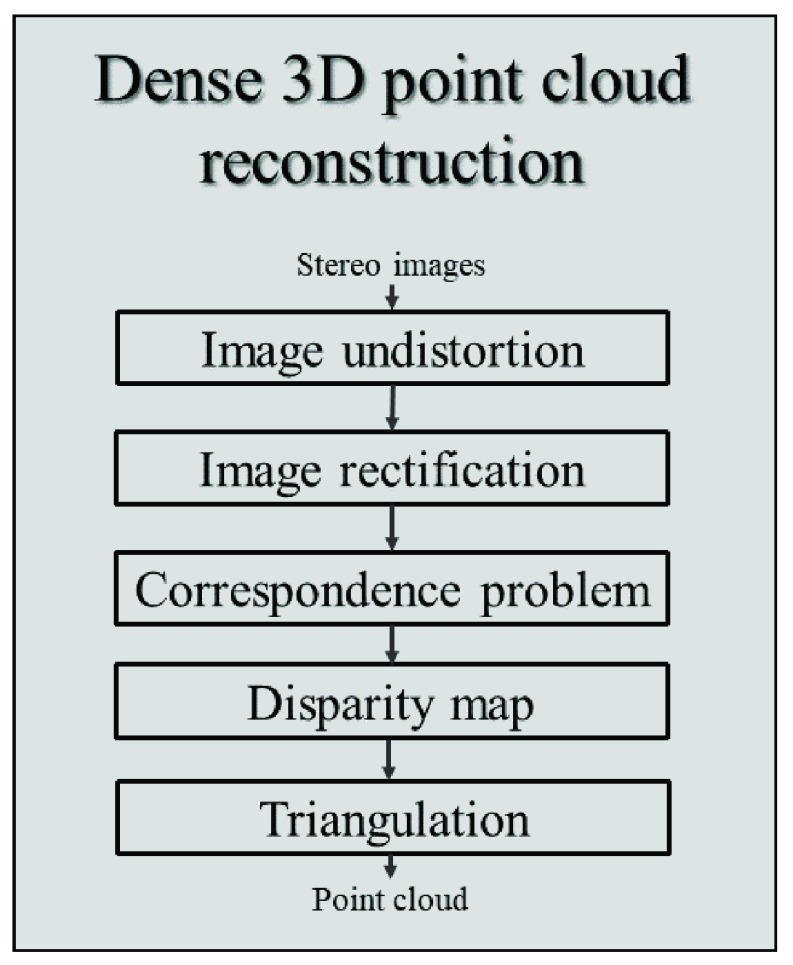
3D reconstruction by stereophotogrammetry. The diagram presents the procedures for obtaining a 3D point cloud from a stereo image pair.

The wound 3D surface is obtained by processing a pair of stereo images through the widely used three-dimensional digital image correlation method. As described in Fig. 3:, this method initially involves undistorting and rectifying the stereo image pair to reduce the complexity of the matching algorithm. Undistortion corrects non-linear deformations caused by the lenses, to allow the modeling of the camera as a pinhole projective system, whereas rectification applies a transformation based on the epipolar geometry to the images that reduces the search for matches of each image pixel to a single row [29]. The points of the disparity map resulting from this process are finally triangulated taking into account the intrinsic and extrinsic parameters obtained from calibration to produce a dense point cloud in 3D space. The resolution of the 3D reconstruction method along the three dimensions $\Delta _{\mathrm {X}}$, $\Delta _{\mathrm {Y}}$, and $\Delta _{\mathrm {Z}}$ are geometrically derived from the intrinsic and extrinsic parameters of the cameras as:

\begin{align*} \Delta _{X}=&\frac {2\ast \mathrm {D}\ast \tan \left ({\frac {FOV_{H}}{2} }\right)}{C_{H}}=0.012~mm; \\ \Delta _{Y}=&\frac {2\ast \mathrm {D}\ast \tan \left ({\frac {FOV_{V}}{2} }\right)}{C_{V}}=0.033~mm; \\ \Delta _{Z}=&\frac {D^{2}}{\mathrm {f}\ast b}=0.83~mm.\tag{3}\end{align*}

where $D$ is the mean working distance, $f$ is the focal length of the cameras, $FOV_{H}$, $FOV_{V}$, $C_{H}$, and $C_{V}$ are the camera parameters as in Table 2, and b is the baseline.

**FIGURE 4.**
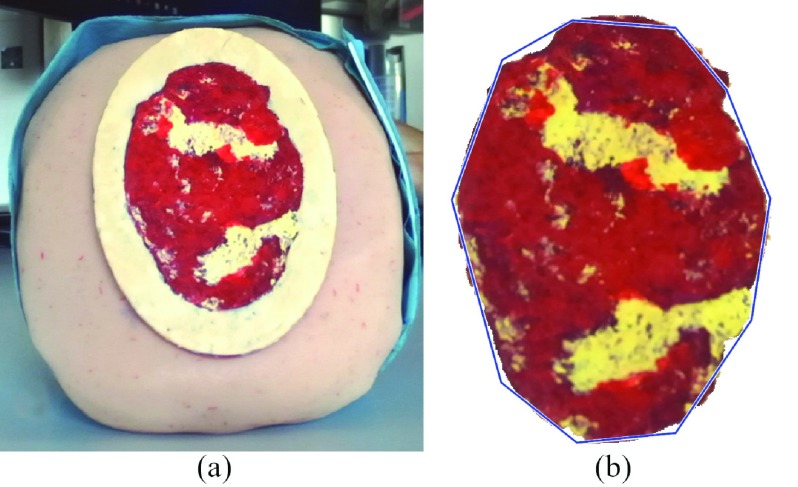
Wound D segmentation steps. a) Wound phantom as captured by the reference camera; b) User manual tracing (blue line), and output of the assisted segmentation algorithm.

**FIGURE 5.**
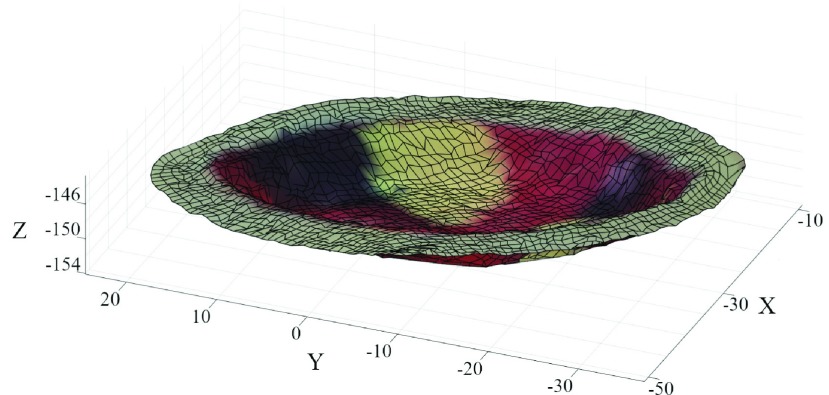
Wound area 3D mesh.

After that, the procedure continues with a semiautomatic 3D point cloud segmentation process performed on the 2D image captured by the reference camera (the left one) remapped on the 3D points. The user is asked to draw a coarse contour with no need to accurately follow the wound boundary. To simplify and speed up the procedure, and reduce the user bias, the contours are automatically adapted to best fit the wound color and texture following the procedure described in [30]. This technique for active contours uses Mumford and Shah [31] segmentation to stop the evolving curve on the desired boundary, offering positive results also in the presence of smooth boundaries. Fig. 4 shows an example of user selection and the resulting segmentation refinement. The segmented image is expanded by 50% from the segmented area to include the surrounding skin, which will serve as a reference for subsequent processing, while the remaining part, which includes the background, is neglected. The segmented area on the 2D image is used to select the corresponding 3D point on the wound. The 3D meshes corresponding to the wound area, with and without the surrounding tissue, are created using a self-organization map (SOM) approach [32]. A SOM is a type of artificial neural network that produces a two-dimensional representation of the input space by using unsupervised machine learning. The representation consists of components called nodes, arranged in a rectangular grid. Each node is given a weight; the map evolves by reducing a distance metric to move weight vectors toward the input data while preserving its topology. In this work, self-organizing maps are employed for reconstruction, so the input data is the 3D point cloud, the map nodes correspond to the output mesh vertices and the weights represent 3D coordinates. The distance metric is the Euclidean distance and it is used in the learning phase to adapt the mesh to the input point cloud. This approach produces a quad-mesh and allows to remove the 3D reconstruction noise and obtain a surface without any holes, producing a mesh as in Fig. 5.

#### 2) Wound Clinical Parameters

In order to obtain reliable clinical parameters, first we will determine the equation of the plane that best fits the skin points of the tissue surrounding the wound. This plane is called $\pi $, p being the generic point of the plane, and is expressed in the Hessian normal form as:

\begin{equation*} \pi:\vec {p}\cdot \vec {n_{0}}-d_{0}=0\end{equation*}

**FIGURE 6.**
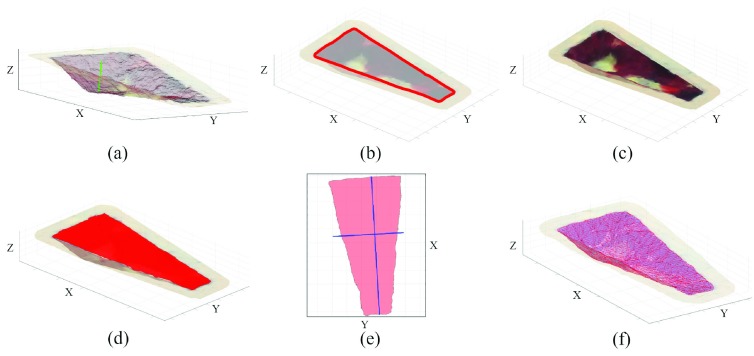
Graphic visualization for geometric parameter computing. a) Wound 3D area is evidenced with respect to the surrounding skin. b) The projected area is shown in red. c) The perimeter is plotted relative to the wound SOM mesh. d) The green line indicates wound depth and is placed in line with the point that produced the depth value. e) The red semi-transparent grid shows the volume of the wound. f) Perpendicular blue lines mark the wound main axes.

*Depth* is derived as the distance between the $\pi $ plane and the farthest point of the wound mesh as:

\begin{equation*} {sup}_{x\in X}(\vec {n_{0}}\cdot \vec {x}-d_{0})\end{equation*}

where x is a point in the wound mesh, X. Fig. 6 (a) shows depth at the deepest point of the mesh.

The software implements the Moore-Neighbor tracing algorithm modified by Jacob’s stopping criteria [33] to find the *perimeter* points of the ulcer in the 2D image of the reference camera. These points are then projected into 3D space through the disparity map to obtain the ulcer perimeter points shown in red in Fig. 6 (b), Xp. Finally, the perimeter is estimated as the sum of the Euclidean distances of contiguous points of the perimeter:

\begin{equation*} \sum \limits _{x\in \mathrm {X}_{p}}^{i} {d(x_{i},\mathrm { }x_{i+1})}\end{equation*}

where d() is the Euclidean distance operator between two points.

The device yields two area values. The former, named *3D area*, is shown in Fig. 6 (c) and is computed directly from the wound mesh as the total area of each quadrilateral of the mesh. Specifically, having named the row index in the node map as $r$ and the column index as $c$, the 3D area is calculated as shown below:

\begin{equation*} \sum \limits _{x\in \mathrm {X}}^{r} \sum \limits _{x\in \mathrm {X}}^{c} \frac {\mathrel {\mathop {\kern 0pt\longrightarrow }\limits _{x_{r,c}x_{r+1,c}}} \ast \mathrm { }\mathrel {\mathop {\kern 0pt\longrightarrow }\limits _{x_{r,c}x_{r,c+1}}} +\mathrel {\mathop {\kern 0pt\longrightarrow }\limits _{x_{r+1,c}x_{r+1,c+1}}} \ast \mathrm { }\mathrel {\mathop {\kern 0pt\longrightarrow }\limits _{x_{r,c+1}x_{r+1,c+1}}} }{2}\end{equation*}

where the * operator computes the cross product. The second value is obtained by projecting the 3D point cloud onto the $\pi $ plane. To simplify operations and speed up the algorithm, the point cloud is rotated so that the $\pi $ plane is parallel to the x-y plane. This simplifies the projection and also reduces the problem to 2D, because the projected points share the same Z coordinate. Finally, the 2D projected points are processed through 2D alpha shape to get a boundary line that encloses all points [34]. The resulting area, shown in Fig. 6 (d) is called *projected area* and was introduced to obtain data comparable with the conventional acetate tracing method [7]. Fig. 6 (e) shows the *main axes* of the wound, also useful for comparison with traditionally acquired manual values.

Main axes are calculated from the projected point cloud by using the principal component analysis. The first principal component is the direction in space, along which projections have the largest variance; this is the largest of the main axes. The second principal component is the direction that maximizes variance in the direction orthogonal to the first, that is the smaller of the main axis.

The *volume*, shown in Fig. 6 (f), is calculated by measuring the region enclosed by the boundary surface produced by applying the 3D alpha shape to the wound mesh [35]. In this process, the $\pi $ plane is used as a constraint to prevent the surface from collapsing inside the wound cavity.

**FIGURE 7.**
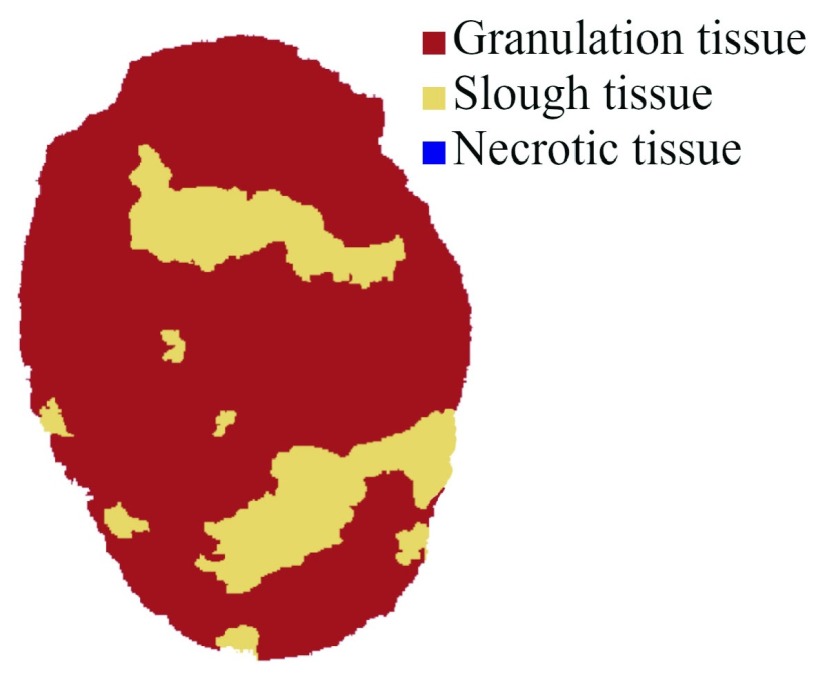
D wound classification. Red: granulation tissue. Yellow: slough. The wound shows no necrotic tissue.

Tissue classification is performed via an image-based procedure that returns the percentage of each tissue type over the total wound 3D surface. The user is asked to select the kind of tissue through a radio button and then pick a seed in a region containing the selected tissue. In order to avoid errors caused by shadows, the successive processing is carried out in the LAB colour space, which allows for the removal of the brightness contribution to the image. Classification is performed on the pre-segmented image using simple linear iterative clustering [36] and is based on the k-mean algorithm, using the user-defined seeds as initial centroids. Fig. 7. shows the result of tissue classification, performed with a k-means classifier.

#### 3) AR Projection of Previous Wound State

The AR module offers the clinician and the patient a tool to directly compare the current wound status with a previous wound status. This functionality relies on two components: a database that stores the geometric and tissue clinical parameters collected at each visit from each patient, and a registration procedure based on a marker placed near the wound. The registration marker currently consists of a 12 $\times\,\,8$ mm square checkerboard.

The registration module first calculates the camera-referenced 3D location of the marker’s fiducials in both the current and previous images. This is achieved through an algorithm that locates the marker in the left and right images, and then triangulates the position of the fiducials to obtain their 3D coordinates. Then, the roto-translation matrix relating to the point sets is derived from a least-squares method [37]. More specifically, given that $\text{p}_{\mathrm {prev}}$ are the marker points in the previous state and $\text{p}_{\mathrm {curr}}$ are the marker points in the current state, the method finds the least square solution for rotation, $\text{R}_{\mathrm {pc}}$, and translation, $\text{T}_{\mathrm {pc}}$, in the equation:

\begin{equation*} p_{curr}\cong R_{pc}~p_{prev}+T_{pc}.\tag{4}\end{equation*}

This roto-translation allows us to align the previous point cloud, loaded from the database, with the current one.

**FIGURE 8.**
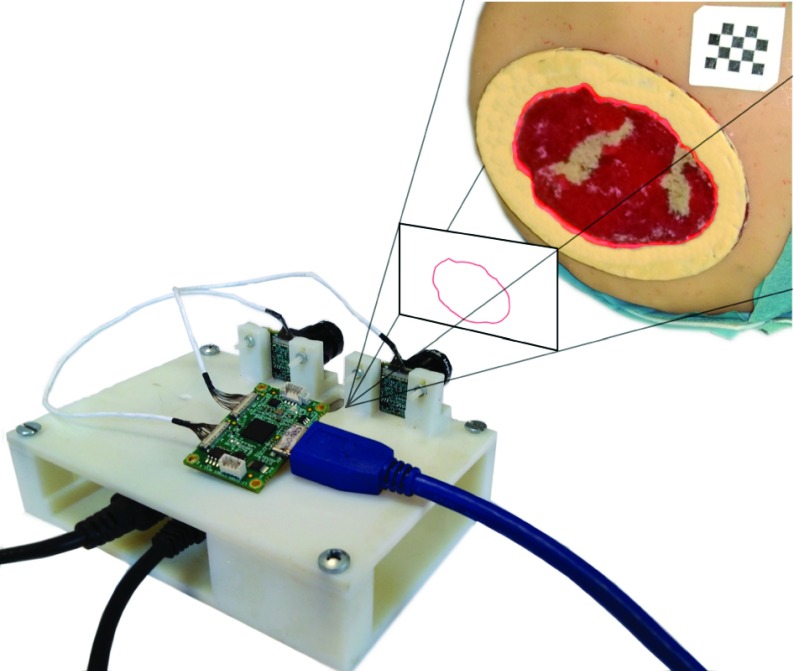
AR projection of previous wound state. The system registers the wound perimeter measured during the previous visit and compares it against the current state through the checkerboard pattern, and then generates an image that the projector uses to visualize the AR content. In this case, the perimeter obtained in the previous visit will overlap the current one, because the ulcer mannequin was not modified.

For the AR functionality, the software projects the previous perimeter points on the image plane of the projector. For AR classification, the procedure is similar: the 3D points of the wound surface are first colored as per the classification, then roto-translated according to $\text{R}_{\mathrm {p-c}}$ and $\text{T}_{\mathrm {p-c}}$, and finally projected onto the projector image plane. The images resulting from this process are transferred to the projector to obtain the AR view as in Fig. 8.

### C. Graphical User Interface Overview

**FIGURE 9.**
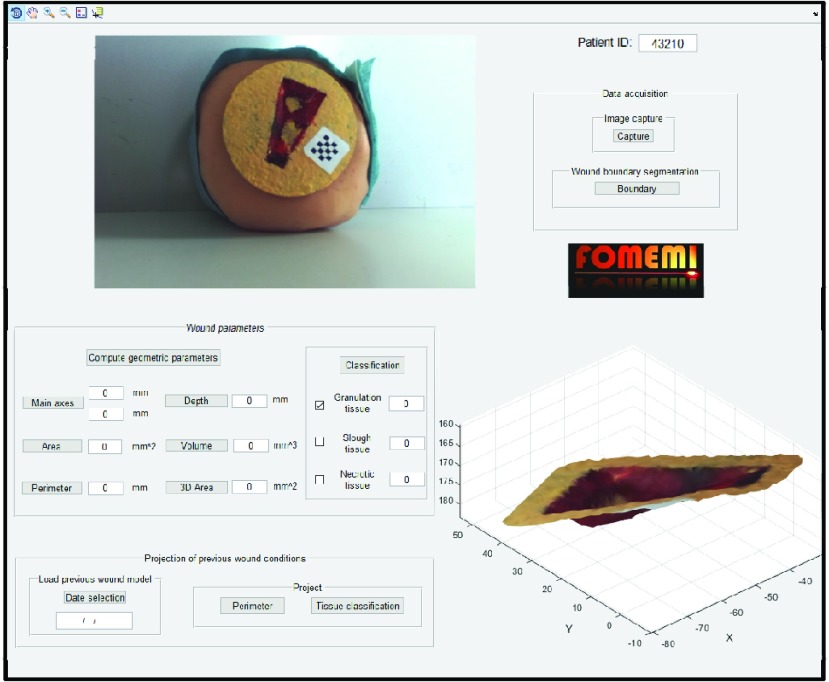
Software GUI. Upper right: The Data acquisition section guides the user from image capture, to segmentation, to visualization of the wound 3D mesh (lower right corner). Bottom left: geometrical parameters and AR projection of previous wound condition.

The GUI has been designed to provide all the above-mentioned features in a simple, fast, and intuitive way. Fig.9 shows an overall view of the GUI. Once the device has been positioned, the user starts the wound healing assessment procedure by pressing the “Capture” button in the Data acquisition module; this will show the acquired images on the screen in real time and allow the user to adjust the device pose, if needed. The image acquired by the reference camera is shown in the upper right corner of the GUI. Then, by pressing the “Boundary” button, a guided procedure allows a fast selection of the wound profiles, then refined by the software. Next, the GUI displays the wound 3D mesh on the screen, that is navigable using the zoom, pan, and rotate buttons. At this point, all the geometric parameters of the ulcer can be calculated by pressing the “Compute geometric parameters” button in the Wound Parameters section. The process takes a few seconds, after which the estimated geometric parameters are loaded in the appropriate fields. If the doctor needs an idea of the processing behind certain values, the parameter of interest can be selected individually to view the processing images in Fig. 6. The classification procedure starts by pressing the “Classification” button in the Wound Parameters module and requires the physician to visually inspect the wound. The AR functionality requires selecting the previous examination for comparison through the “Date selection” button of the Load Previous Wound model. Then, by pressing the “Perimeter” or “Tissue Classification” buttons, the software registers the previous models with the current one by using the marker, and the corresponding AR information is projected.

### D. Device Assessment

The system has been tested by novice users to assess accuracy, precision, and inter-rater reliability in the estimation of geometric parameters. Repeatability was then analyzed to assess the agreement between successive measurements under the same conditions. Finally, the accuracy of the AR projection was assessed.

**FIGURE 10.**
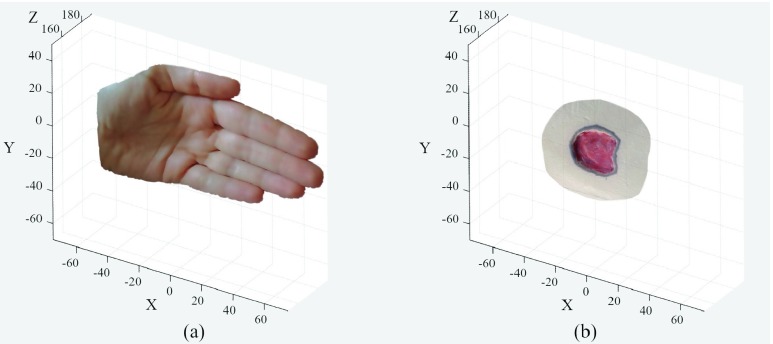
Device clinical transaction. a) 3D mesh from tests on a healthy hand. b) 3D mesh from mannequin ulcer by VATA (Arterial Insufficiency Leg).

Tests on healthy human limbs and on an ulcer mannequin (Arterial Insufficiency Leg mannequin by VATA) were carried out to prove the clinical translation of the system. The device produced 3D meshes of human hand, arm and leg portions and mannequin ulcers, also returning, in this case, wound morphological parameters. Fig. 10 shows the 3D meshes resulting from the tests performed on the hand and mannequin ulcer.

#### 1) Geometrical Parameters

**FIGURE 11.**
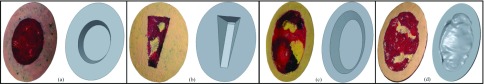
Wound models printed with Stratasys Object30 Prime and corresponding CAD model designed with PTC Creo 3.0: (a) Wound A (b) Wound B (c) Wound C (d) Wound D.

Four models simulating a healing wound were designed with the PTC Creo 3.0 software to provide real reference values for measurements. A Stratasys Objet30 Prime 3D printer allowed for a rapid prototyping of the wound models with a 600 dpi X-Y resolution, 1600 dpi Z resolution and 0.1 mm precision. The phantoms were created with Stratasys VeroClear and VeroBlack materials, with a 16-micrometer thickness of the printing layer, and they were covered with a thin layer of enamel paint to simulate granulation, slough, and necrotic tissues. Fig. 11 shows the wound phantoms and their corresponding CAD models. Wounds with a great variety of sizes and shapes are reported in the clinical scientific literature [38], therefore we designed completely different realistic models ranging from a small circular ulcer, which simulates a healing ulcer (wound A), to a completely irregular ulcer in both shape and depth (Wound D). Wound A mostly consists of granulation tissue; Wound B has a trapezoidal shape and presents a gradually increasing depth from 0 mm to 7.5 mm; Wound C has an elliptical shape and shows granulation, exudate, and necrotic tissue; Wound D has an uneven depth up to 5.4 mm, irregular shape and slough and granulation tissue. Table 3 shows all the geometric parameters derived from the CAD models and used as gold standard for comparison with the parameters measured by the device.

**TABLE 3:** Geometric Parameters Calculated in PTC CREO for Each Wound Model

Wound model	3D Area (mm2)	Projected Area (mm2)	Perimeter (mm)	Depth (mm)	Volume (mm3)	Major Axis (mm)	Minor Axis (mm)
A	744.50	706.85	94.24	3	1385	30	30
B	1070.08	825	133.33	7.50	2679	50	25
C	1413.83	1178.10	127.63	5	4444.60	50	30
D	2077	1841	163.73	5.40	4601	60	40

The device was tested by 11 users without any previous familiarity with our system. Each user received a 10 minute training on the features and acquisition modes of the GUI. Specifically, users were trained to set a working distance between 15 cm and 35 cm and a device orientation, which must be as perpendicular as possible to the wound bed in order to maximize the wound size in the images (to be able to scan the entire wound bed without occlusions).

Each user tested the device on each of the wound models, for a total of 44 tests. The collected data were used to assess accuracy, precision, and inter-rater reliability. The working distance was also recorded to evaluate compliance with the acquisition methods. Accuracy was estimated as the difference from the reference value according to (1). Precision was determined as the standard deviation of measurements according to (2). Inter-rater reliability was determined by comparing the measurements of the same wound taken by different users through the intraclass correlation coefficient (ICC). In particular, the ICC(2,1) coefficient [39] was used: users have been considered to be representative of a larger population of similar users, so no familiarity with the device was required. In addition to geometric parameters, the measurement time was also recorded as the interval from the initial acquisition by the stereo-cameras to the data display.

A final set of tests was performed for the evaluation of repeatability. The procedure was repeated 10 subsequent times by the same user, in the same location. Following the guidelines of [40], a repeatability value for each parameter was computed from the one-way analysis of variance (ANOVA).

#### 2) AR Projection

**FIGURE 12.**
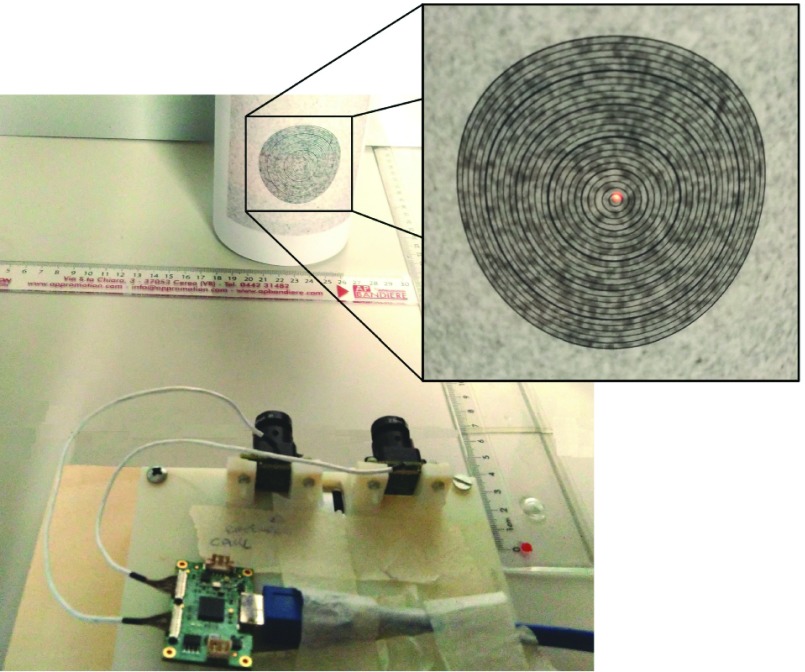
Experimental setup for projection accuracy evaluation.

An image target consisting of 1-mm radius increment concentric circles was created to quantitatively evaluate the error in the re-projection of the wound. As shown in Fig. 12, the image was applied to a cylindrical object, so that the target points lay at different depths from the cameras. The target was then acquired and the outside circle manually segmented following the guided procedure described in section II-C until the 3D model was obtained. Subsequently, a variation to the algorithm consisting in the calculation of the median of the point cloud was introduced, and the median point was projected on the target. The inner circles were then used as reference to evaluate the distance between the projected center and the target center. If the projected point fell across a circle, as in Figure 12, the radius of the enclosed circle was taken as the projection error. If it fell between two rings, the average radius of these rings was taken as the projection error. The projection error was then determined as the distance between the projected center and the target center.

The tests were repeated with consecutive steps of 2.5 mm inside the device working space and the average value from 3 measurements was reported for each distance.

## III. Results

### A. Camera Stereo Calibration

**FIGURE 13.**
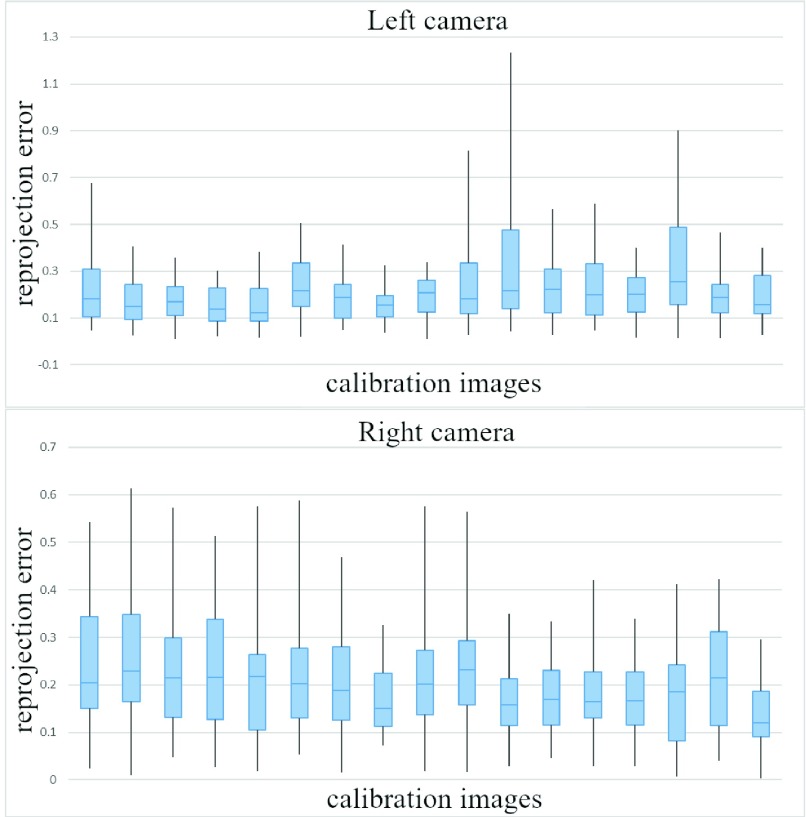
Intrinsic calibration evaluation. The re-projection error is expressed in pixels. Blue boxes represent the upper and lower quartiles of re-projection errors. Black lines denote the minimum and maximum error values.

Intrinsic calibration parameters for both the left and right cameras are listed in Table 4 with a 95% confidence interval. The re-projection error is the standard method to evaluate intrinsic calibration accuracy; it corresponds to the distance between a checkerboard corner in a calibration image and the respective world point projected into the same image. Fig. 13 shows the boxplots associated with re-projection errors in the left and right cameras. The overall mean re-projection errors for the left and right cameras were 0.21 px and 0.20 px, respectively.

**TABLE 4:** Intrinsic Calibration Parameters

	Left camera		Right camera	
Focal length (px)	1603 ± 24	1605 ± 24	1650 ± 20	1649 ± 20
Principal point (px)	1089 ± 8	629 ± 8	1117 ± 6	636 ± 8
Radial distortion	−0.171 ± 0.004	0.028 ± 0.002	−0.184 ± 0.004	0.034 ± 0.002

Intrinsic calibration parameters for left and right cameras. Each value is associated with the 95% confidence interval.

**FIGURE 14.**
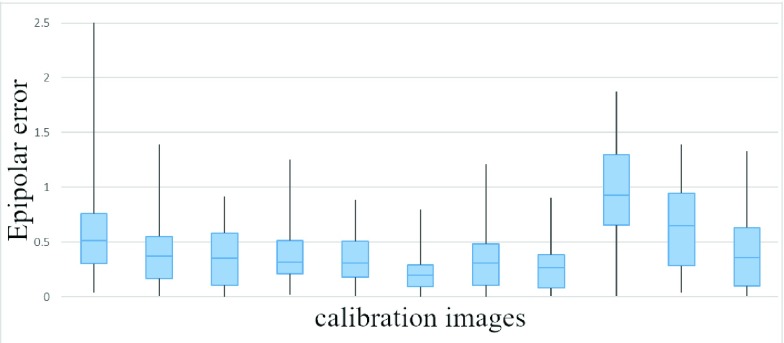
Extrinsic calibration evaluation. The epipolar error is in pixels. Blue boxes represent the upper and lower quartiles of epipolar errors. Black lines denote the minimum and maximum error values.

The epipolar error was used to assess extrinsic calibration quality. In a stereo camera configuration, a point in one camera view must fall along a single line in the other camera view. This line is the epipolar line of that point, and its distance from its corresponding point in the other camera image is the epipolar error. The boxplots in Fig. 14 report statistics for the epipolar errors produced by each checkerboard point for each calibration image; the average error is 0.45 px.

### B. Camera-Projector Calibration

**FIGURE 15.**
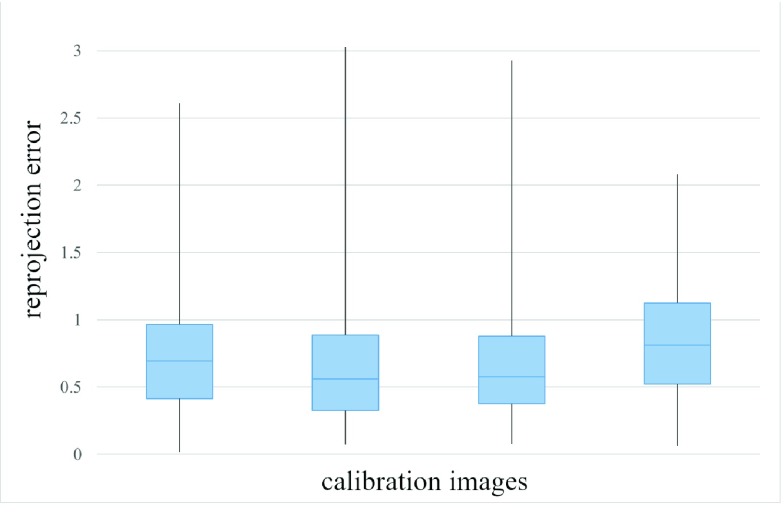
Camera-projector calibration evaluation. The re-projection error is in pixels. Blue boxes represent the upper and lower quartiles of re-projection errors. Black lines denote the minimum and maximum error values.

The quality of the calibration between the camera and the projector is indicated by the re-projection error in Fig. 15. The error refers to the difference in pixels between the AR checkerboard points in the image and the re-projection of the checkerboard 3D points to the same camera image. The latter are determined by the intersection of the plane identified by the real checkerboard and the rays from the digital checkerboard corners in the image plane of the projector.

### C. Geometrical Parameters

All users took measurements within the workspace with an average time of 1min 5s ± 18s. Table 5 lists the accuracy and precision values for each geometric parameter. Tables 6 and 7 respectively report the results for inter-rater variability and measurement repeatability coefficients.

**TABLE 5:** Accuracy and Precision of the Geometric Parameters

Parameter	3D Area	Projected Area	Perimeter	Major axes	Minor axes	Depth	Volume
Accuracy (%)	3.53	3.69	1.43	3.51	0.92	2.23	−2.04
Precision (%)	1.16	0.58	0.75	0.79	1.16	2.18	2.28

**TABLE 6:** Intraclass Correlation Coefficient for Geometric Parameters

Parameter	3D Area	Projected Area	Perimeter	Major axes	Minor axes	Depth	Volume
ICC	0.999	1	0.998	1	0.996	0.996	0.996

**TABLE 7:** Repeatability Coefficients for Geometric Parameters

Parameter	3D Area	Projected Area	Perimeter	Major axes	Minor axes	Depth	Volume
Repeatability	0.9998	0.9997	0.9981	0.993	0.9972	0.993	0.994

### D. AR Projection

**FIGURE 16.**
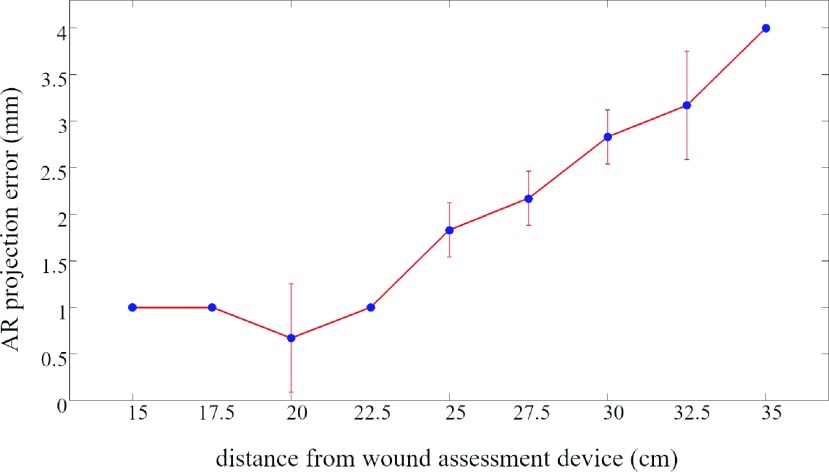
Projection error. Blue points denote the mean error, whereas error bars represent the error standard deviation. No error bar is plotted when standard deviation is less than 0.01 mm.

Tests to evaluate the AR projection error yielded the results shown in Fig. 16 for distances within the working area. The lowest error occurred at 20 cm from the device and it increased by moving towards the workspace boundaries.

## IV. Discussion

Our camera stereo calibration achieved sub-pixel accuracy. This ensures high-quality stereo reconstruction, which provides us with a solid basis for the calculation of reliable parameters [41]. In fact, the accuracy and precision of geometric parameter measurements are similar and mostly better compared to currently marketed devices. Parameters more prone to error, such as depth and volume, are calculated with an accuracy of about two percentage points off with respect to the 4% depth accuracy of the Silhouette device and to the 3.46% volume accuracy of eKare [14], [15]. There are no terms of comparison available for the projected area and the main axes, which, however, show good accuracy values and an average precision of 0.8%. The measurement time of about one minute considerably speeds up the inaccurate and unpleasant traditional procedure for wound healing assessment, leaving the clinician more time to determine the best specific treatment for the patient. The short measurement time also allows clinicians to use AR without extending the visit, which improves doctor-patient communication. The AR projection reveals a working distance of 20 cm as the optimal distance in terms of projection error, which is 0.67 mm. The error reaches its maximum value at 35 cm. This behavior may be due to inaccuracies in the extrinsic camera-projector calibration, which, however, showed a low re-projection error, so further work is needed to achieve consistent accuracy in the working area. ICC values indicate excellent inter-rater reliability according to the ranges given in [39], but for a proper assessment we need to increase the sample size. ICC values close to 1 suggest that bias sources imputable to the user are not relevant, and the work done to refine the segmentation of wound boundaries has been successful.

All users took measurements within the workspace, so the other source of bias relates to the device orientation only. Based on these considerations and given that all the users in the study used the device for the first time, we can state that the device is easy-to-use and gives excellent results in terms of accuracy and precision. The HW components we used have been selected on the basis of the following lines of reasoning: a mini projector can offer an AR view directly on the patient; at least one camera in the visible spectrum is required to allow the physician to perform tissue classification; to automatically obtain wound geometric parameters, 3D scanning functionalities are required and, in theory, a projector and a camera are enough to scan the wound by using structured light or phase shift techniques [42], [43]. In any case, to obtain a good model resolution, both these approaches require to capture more than one image, while keeping the relative position between the limb and the fixed scanning system (camera and projector). Moreover, the resolution of 3D systems based on a single camera and a projector is strictly related to the projector resolution, which is usually low for mini projectors compared to the camera resolution. Lastly, obtaining a wound 3D model by projecting on a highly heterogeneous wound area could cause contrast issues affecting the accuracy of the reconstruction, whereas, in stereo camera systems, texture and color heterogeneity is associated with a good 3D accuracy. For this reason, we introduced a second camera and used a stereo matching technique to obtain information about depth.

## V. Conclusion

The purpose of this work was to create a practical and non-invasive device for wound healing assessment, while providing augmented reality functionalities to facilitate doctor-patient communication with a direct feedback on the condition of the wound.

The healing process is assessed by using stereo cameras, which simultaneously yield tissue classification and accurate 3D wound reconstruction. High-resolution 3D models are created based on the natural wound heterogeneity and skin texture without compromising the ease of use of the device. The tests produced accurate and precise values for the geometric parameters of the wound in a short time and in a totally non-invasive manner. Users who are familiar with the device take about 42s to complete the assessment procedure. Accuracy and precision for linear parameters are 2% and 1.7%, respectively; for surface and volume they are 1.2% and 1.3%, respectively. This shows that very good estimates are given of the two parameters, which are subject to major errors even in devices that focus more on accuracy than on time. The evaluation of inter-rater reliability produced excellent results, suggesting that user-related sources of variability are minimal.

With the same objective of producing a user-friendly device, we chose a projector to provide AR info, leaving the surgeon’s hands and vision free. The assessment of the accuracy of AR projection produced an error at the optimal distance of 0.67 mm ± 0.58 mm.

We can conclude that for an accurate and complete 3D wound healing assessment device featuring AR functionalities to be produced, two cameras in the visible domain and a mini projector are needed.

To the best of our knowledge, this is the first system capable to obtain a non-contact measurement of wound clinical parameters paired with AR projection to easily and intuitively follow the wound evolution.

The above-mentioned technical validation paves the way for the next step: a clinical validation on human patients.
